# Venetoclax combined with daunorubicin and cytarabine (2 + 6) as induction treatment in adults with newly diagnosed acute myeloid leukemia: a phase 2, multicenter, single-arm trial

**DOI:** 10.1186/s40164-023-00409-y

**Published:** 2023-05-12

**Authors:** Xiaohui Suo, Fang Zheng, Dongmei Wang, Liyun Zhao, Jie Liu, Ling Li, Zhihua Zhang, Congcong Zhang, Yinling Li, Sisi Yang, Xuemei Zhao, Rui Shi, Yan Wu, Zongjiu Jiao, Jiaojie Song, Ling Zhang, Xinxiao Lu, Linyu Yuan, Sifeng Gao, Jilei Zhang, Xingli Zhao, Guanchen Bai, Kaiqi Liu, Yingchang Mi

**Affiliations:** 1 Department of Hematology, Handan Central Hospital, Handan, Hebei China; 2 Department of Hematology, Baiyun Hospital Affiliated to Guizhou Medical University, Guiyang, Guizhou China; 3 Department of Hematology, Harrison International Peace Hospital, Hengshui, Hebei China; 4 Department of Hematology, People Hospital of XingTai, Xing Tai, Hebei China; 5 Department of Hematology, Sinopharm Tongmei General Hospital, Datong, Shanxi China; 6 Department of Hematology, Inner Mongolia People’s Hospital, Huhehaote, Neimenggu China; 7 Department of Hematology, The Affiliated Hospital of Chengde Medical College, Chengde, Hebei China; 8 Department of Hematology, Oncology Center, Tianjin People’s Hospital, No. 190 Jieyuan Road, Hongqiao District, Tianjin, China; 9 Department of Hematology, The Affiliated Tai’an City Central Hospital of Qingdao University, Taian, Shandong China; 10 State Key Laboratory of Experimental Hematology, Institute of Hematology & Blood Diseases Hospital, National Clinical Research Center for Blood Diseases, Chinese Academy of Medical Sciences & Peking Union Medical College, Tianjin, China; 11 Institute of Hematology and Blood Diseases Hospital, CAMS & PUMC, Tianjin, China

**Keywords:** Venetoclax, DA, Induction treatment, Acute myeloid leukemia

## Abstract

**Background:**

Venetoclax (Ven) combined with intensive chemotherapy was proven effective in the management of acute myeloid leukemia (AML). However, the severe and prolonged myelosuppression remains a concern to worry about. To explore more appropriate combination regimens, we designed Ven combining daunorubicin and cytarabine (DA 2 + 6) regimen as induction therapy, aimed to evaluate the effectiveness and safety in adults de novo AML.

**Methods:**

A phase 2 clinical trial was performed in 10 Chinese hospitals to investigate Ven combined with daunorubicin and cytarabine (DA 2 + 6) in patients with AML. The primary endpoints were overall response rate (ORR), comprising of complete remission (CR), complete remission with incomplete blood cell count recovery (CRi), and partial response (PR). Secondary endpoints included measurable residual disease (MRD) of bone marrow assessed by flow cytometry, overall survival (OS), event-free survival (EFS), disease-free survival (DFS), and the safety of regimens. This study is a currently ongoing trial listed on the Chinese Clinical Trial Registry as ChiCTR2200061524.

**Results:**

Overall, 42 patients were enrolled from January 2022 to November 2022; 54.8% (23/42) were male, and the median age was 40 (range, 16–60) years. The ORR after one cycle of induction was 92.9% (95% confidence interval [CI], 91.6–94.1; 39/42) with a composite complete response rate (CR + CRi) 90.5% (95% CI, 89.3–91.6, CR 37/42, CRi 1/42). Moreover, 87.9% (29/33) of the CR patients with undetectable MRD (95% CI, 84.9–90.8). Grade 3 or worse adverse effects included neutropenia (100%), thrombocytopenia (100%), febrile neutropenia (90.5%), and one mortality. The median neutrophil and platelet recovery times were 13 (5–26) and 12 (8–26) days, respectively. Until Jan 30, 2023, the estimated 12-month OS, EFS, and DFS rates were 83.1% (95% CI, 78.8–87.4), 82.7% (95% CI, 79.4–86.1), and 92.0% (95% CI, 89.8–94.3), respectively.

**Conclusion:**

Ven with DA (2 + 6) is a highly effective and safe induction therapy for adults with newly diagnosed AML. To the best of our knowledge, this induction therapy has the shortest myelosuppressive period but has similar efficacy to previous studies.

**Supplementary Information:**

The online version contains supplementary material available at 10.1186/s40164-023-00409-y.

## Introduction

Acute myeloid leukemia (AML) is the most common type of acute leukemia in adults and carries a poor prognosis. For patients who are eligible for intensive chemotherapy, the standard induction therapy is still anthracycline(s) combined with cytarabine (DA 3 + 7), which results in complete remission (CR) rates of 60%–80% and long-term overall survival (OS) rate < 50% [1–5]. Other induction therapies that added purine analogs improved outcomes in AML [6]. However, the prognosis of adults with AML remains discouraging.

Venetoclax (Ven), an oral Bcl-2 inhibitor, selectively binds to Bcl-2 to release proapoptotic proteins in tumor cells [7]. Numerous clinical trials have shown that the combination of Ven with azacytidine (AZA), decitabine (DEC), and low-dose cytarabine can significantly improve the overall response rate (ORR) and OS of newly diagnosed AML patients who cannot tolerate intensive chemotherapy [8–11]. Hence, these therapeutic regimens have rapidly become first-line therapy for such patients. For patients who can tolerate chemotherapy, Ven combined with intensive chemotherapy (DA, reduced-dose IA, CLIA, FLAG-IDA) improved CR rate and increased the minimal residual disease (MRD) negative rate [12–15]. 97% ORR was reported in de novo AML patients treated with Ven combing IA (2 + 5). In another study using Ven plus FLAG-IDA, the ORR was 97% with 96% MRD (−) CR (flow cytometry) in newly diagnosed AML. Similar efficacy was observed in Ven plus CLIA. However, Ven combined with intensive chemotherapy also resulted in prolonged myelosuppression without reducing transplantation rates. The median time to blood cell count recovery during induction period in responding patients was approximately 4 weeks with both FLAG-IDA and CLIA regimens, and the median treatment courses was two [14, 15]. Therefore, optimizing the combination of Ven with chemotherapy is essential to improve the tolerance and long-term survival.

To explore an optimal combination of Ven with chemotherapy as frontline induction regimen for AML, we conducted a multicenter, single-arm, phase 2 trial to investigate the safety and effectiveness of Ven combined with daunorubicin and cytarabine (DA 2 + 6) as induction treatment for newly diagnosed AML in adults.

## Methods

### Study design and participants

This study was an investigator-initiated, phase 2, multicenter, single-arm trial at 10 hospitals in China (additional details are provided in the Additional file 1: Appendix).

The inclusion criteria were patients aged 16–60 years with de novo AML according to the World Health Organization diagnostic criteria (2016); Eastern Cooperative Oncology Group performance status of 0–2; and adequate renal, hepatic, and cardiac functions. The exclusion criteria included patients with acute promyelocytic leukemia, secondary leukemia, and a history of myelodysplastic or myeloproliferative neoplasms. Patients who had received previous treatment, pregnant and/or lactating, unable to sign the informed consent form were excluded. All inclusion and exclusion criteria are included in the Additional file 3.

The study procedures were conducted following the principles of the Declaration of Helsinki and approved by the Health Human Research Ethics Committee from the Handan Central Hospital, Handan, Hebei, China. Written informed consent was acquired from the patients or their parents/lawful guardians before participating.

### Procedures

**Fig. 1:**
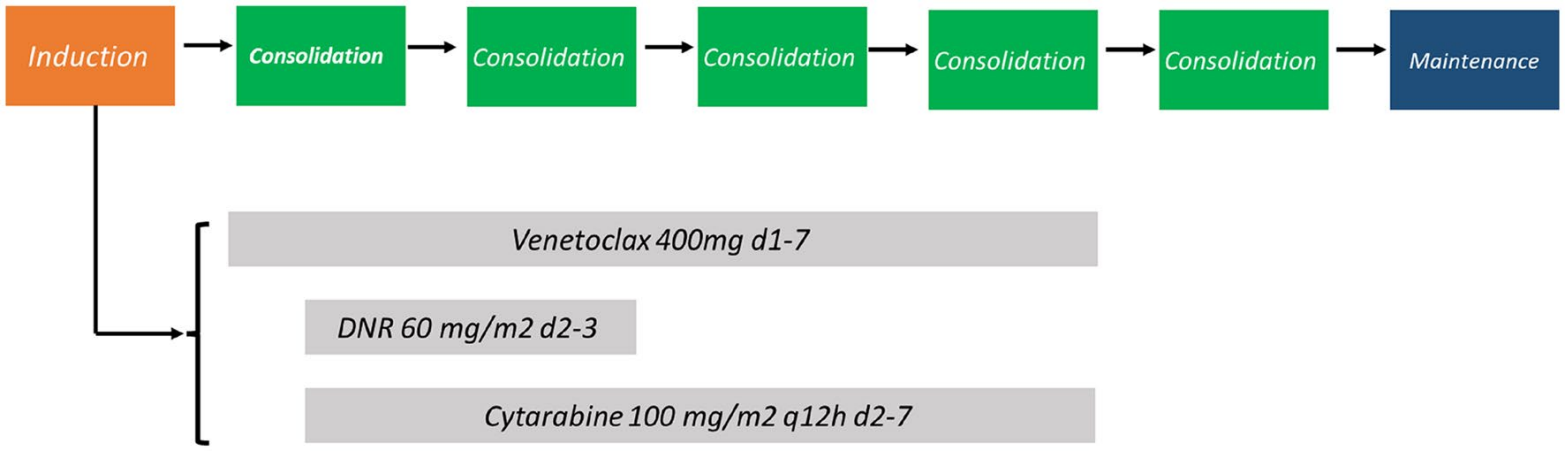
Treatment schema *DNR* daunorubicin

The treatment schema is summarized in Fig. 1. The induction regimen consisted of oral Ven (400 mg, Days 1–7), intravenous daunorubicin (60 mg/m^2^, Days 2–3), and cytarabine (100 mg/m^2^/q12 h, Days 2–7). Patients with significantly elevated white cell counts, hydroxyurea and cytarabine were permitted for cytoreduction before induction therapy to decrease the possibility of tumor lysis syndrome until the white cell count (WBC) was ≤ 30 × 10^9^/L. Patients receiving potent CYP3A inhibitors, such as voriconazole and posaconazole, Ven reduced to 100 mg. During neutropenic phase after induction therapy, granulocyte colony-stimulating factor was permitted. Antifungal prophylaxis with voriconazole and posaconazole was administered during neutropenic episodes based on the experience of each center.

Patients who achieved partial response (PR) after induction therapy underwent reinduction therapy with the same regimen. Patients with no response (NR) after one cycle of induction therapy or who did not reach CR or CRi following two cycles of induction therapy were unenrolled from the study and received other salvage treatments.

After achieving CR or CRi, patients who were candidates for allogeneic hematopoietic stem-cell transplantation (allo-HSCT) according to ELN guidelines and Chinese guidelines for the diagnosis and treatment of adult AML (2017) [16] were recommended to receive allo-SCT. Patients who did not need or could not undergo allo-HSCT received consolidation and maintenance therapy.

In previous reports, Ven combined with chemotherapy resulted in a significantly increased duration of myelosuppression [13–15]. Our previous exploratory research compared high-dose cytarabine (Ara-c 3 g/m^2^/q12, Days 1, 3, and 5) with Ven combined with intermediate-dose cytarabine (Ven 400 mg on Days 1–7, cytarabine 1 g/m^2^/q12 h Days 2–4), and the results showed a similar duration of myelosuppression and comparable safety and efficacy (see Additional file 2). Hence, our consolidation therapy included two cycles of Ven combined with intermediate-dose cytarabine (Ven 400 mg on Days 1–7, cytarabine 1 g/m^2^/q12 h Days 2–4) and three cycles dose-reduced chemotherapy with DA (daunorubicin 60 mg/m^2^, Days 1–2 and cytarabine 100 mg/m^2^/q12h, Days 1–5), 2 cylces HA (homoharringtonine 2 mg/m^2^, Days 1–5 and cytarabine 100 mg/m^2^/q12h, Days 1–5).

As most patients might not accept allo-HSCT, we designed maintenance therapy regimen after completing consolidation treatment. Maintenance therapy involved Ven combined with AZA, danazol (DNZ) and thalidomide (THD) (Ven 200 mg, Days 1–7; AZA 100 mg, Days 1–5; DNZ 200 mg po bid, Days 8–28; THD 100 mg po qd, Days 8–28). Maintenance was permitted on Days 1–28 every month for at least six cycles in patients not proceeding to stem-cell transplantation. After stopping Ven and AZA, patients continued the DNZ and THD maintenance therapy for up to 3 years unless relapse or intolerance occurred.

### Efficacy and safety

To evaluate the response to treatment, the bone marrow was evaluated on Days 28 to 35 after induction with blood count recovery. Morphological and measurable residual disease (MRD) assessments of bone marrow were conducted. MRD was analyzed using multi-parameter flow cytometry with a sensitivity of 0.01–0.1% [17]. Quantitative polymerase chain reaction (qPCR) was used for MRD assessments if the patient presented with special fusion transcripts, such as CBFB-MYH11 and RUNX1-RUNX1T1 (AML1-ETO) [18].

Adverse events were monitored and classified according to the Common Terminology Criteria for Adverse Events of the National Cancer Institute, version 5.0 [19].

### Outcomes

The primary endpoint was the ORR, comprising of the CR, CRi, PR, and MLFS, after one cycle induction treatment as per the modified International Working Group response criteria for AML [20]. CR was defined as bone marrow blasts < 5% and without of blasts with Auer rods, absence of circulating blasts, absence of extramedullary disease, ANC ≥ 1.0 × 10^9^/L (1000/μL), and platelet count ≥ 100 × 10^9^/L (100,000/μL). CRi was defined as meeting all CR criteria except for neutropenia < 1.0 × 10^9^/L (1000/μL) or thrombocytopenia < 100 × 10^9^/L (100,000/μL). PR was defined as meeting all hematologic criteria of CR, a decrease in the bone marrow blast percentage to 5–25%, and a decrease in the pretreatment bone marrow blast percentage by at least 50%. MLFS was defined as bone marrow blasts < 5%, absence of blasts with Auer rods, absence of circulating blasts, absence of extramedullary disease, and no hematologic recovery needed.

The secondary endpoints were MRD detected by flow cytometry after one cycle of induction therapy, OS, event-free survival (EFS), disease-free survival (DFS), and the safety of the first induction regimen. OS was defined from day 1 of registration to the date of death from any cause or censorship at the last follow-up. EFS was defined as the duration from treatment initiation to the occurrence of induction failure, relapse, or death, whichever came first. DFS was defined as the duration from the date of disease remission to the occurrence of relapse or censorship at the last follow-up. Early death was defined as death within 30 days of chemotherapy.

### Statistical analysis

The calculation of the sample size is shown in the Additional file 4. Statistical analyses were performed with SPSS (v. 25.0). The exact binomial method was employed to calculate the response rate with 95% credible intervals. OS, EFS, and RFS rates were extracted based on Kaplan–Meier estimates with 95% confidence intervals (CI) and were compared through the log-rank test. This trial is ongoing and is registered with the Chinese Clinical Trial Registry, ChiCTR2200061524.

## Results

**Fig. 2:**
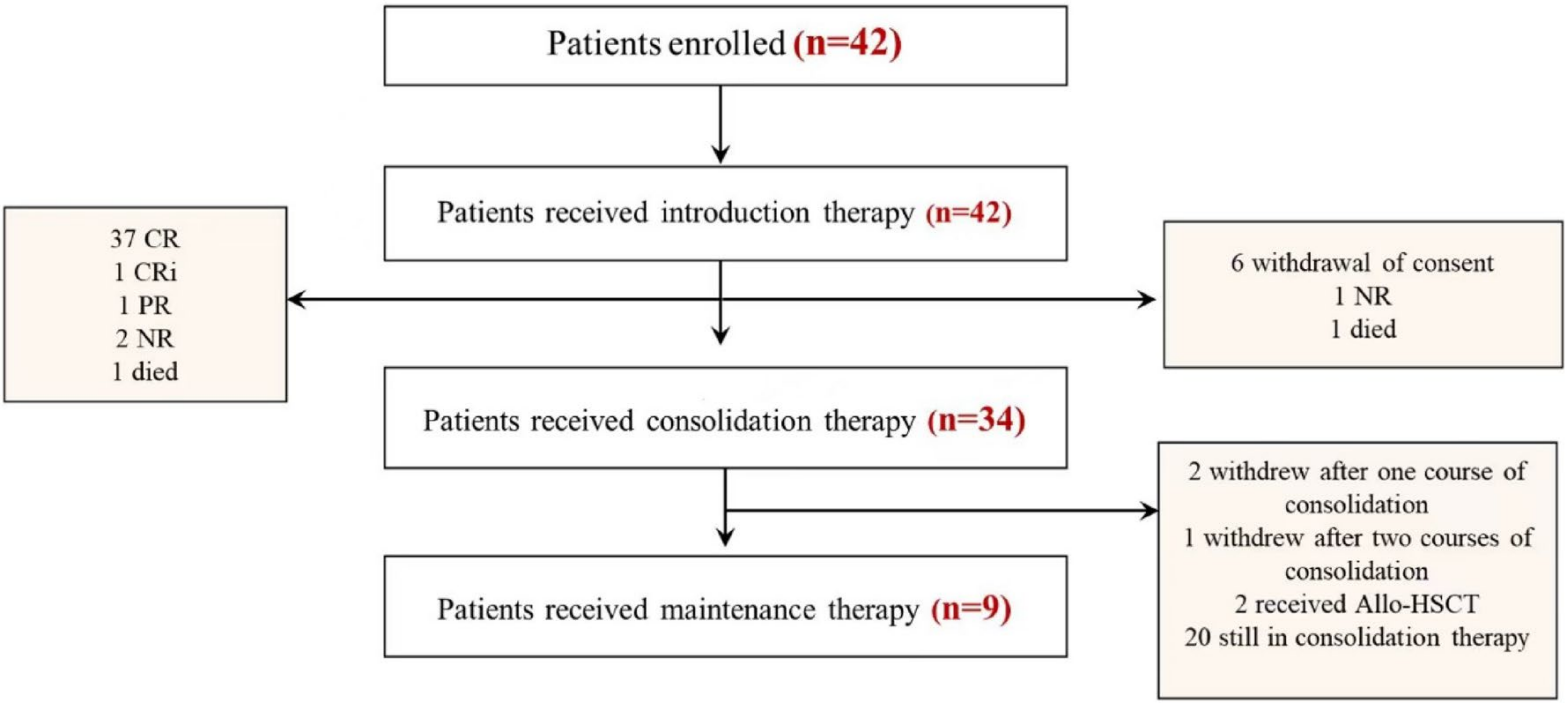
Trial profile *Allo-HSCT* Allogeneic–hematopoietic stem-cell transplantation *CR* complete remission *CRi* complete remission with incomplete blood cell count recovery *PR* partial response *NR* no response

Overall, 42 patients with newly diagnosed AML were enrolled between January 1, 2022, and November 30, 2022 (Fig. 2). Baseline characteristics of the patient are summarized in Table 1. The median age was 40 (range, 16–60) years, and 23 (54.8%) patients were male. The median WBC was 8.0 (range, 0.29–201.00) × 10^9^/L. Among the cohort, 14 (33.3%) patients had infection before induction therapy, 6 (14.3%) received CYP3A4 inhibitors (4 voriconazole and 2 posaconazole) during Ven induction therapy, and 3 (7.14%) were administered oral Ven (200 mg) on Day 1. According to the European Leukemia Network prognostic group (2022), 17 (40.5%), 6 (14.3%), and 19 (45.2%) patients were considered to belong to the favorable, intermediate, and adverse groups, respectively. Favorable-risk cytogenetics (core-binding factor) were detected in 11 patients; 10 patients with RUNX1-RUNXT1/t (8;21), 1 with CBFB/MYH11/inv (16). Gene mutations included of FLT3-ITD (30.9%), DNMT3A (21.4%), NPM1 (16.7%), TET2 (14.3%), RUNX1 (14.3%), BCOR (14.3%), KIT (14.3%), RAS (14.3%), CEBPA (B-zip) (11.9%), FLT3-TKD (9.5%), IDH2 (9.5%), PHF6 (9.5%), WT1 (9.5%), ASXL1 (7.1%), IDH1 (7.1%), SRSF2 (4.8%), PTPN11 (7.1%), JAK2 (4.8%), EZH2 (2.4%), STAG2 (2.4%), and CSF3R (2.4%). (Table 1).

**Table 1:** Baseline characteristics of the 42 AML patients

Characteristic	
Total number of patients	42
Median age, years (range)	40 (16–60)
Gender	
Male	23 (54.8%)
Female	19 (45.2%)
ECOG	1 (0–1)
Baseline WBC (× 10^9^/L) Median (IQR)	8.0 (0.29–201)
Baseline platelet count, (× 10^9^/L) Median (IQR)	37 (6–812)
ELN 2022 risk group	
Favorable	17 (40.5%)
Intermediate	6 (14.3%)
Adverse	19 (45.2%)
ELN 2017 risk group	
Favorable	18 (42.9%)
Intermediate	10 (23.8%)
Adverse	14 (33.3%)
Cytogenetic risk category	
Favorable	11 (26.2%)
Intermediate	22 (52.4%)
Adverse	8 (19%)
No mitosis	1 (2.4%)
Selected molecular mutations	
FLT3-ITD	13 (30.9%)
DNMT3A	9 (21.4%)
NPM1	7 (16.7%)
TET2	6 (14.3%)
RUNX1	6 (14.3%)
BCOR	6 (14.3%)
KIT	6 (14.3%)
RAS	6 (14.3%)
CEBPA (B-zip)	5 (11.9%)
FLT3-TKD	4 (9.5%)
IDH2	4 (9.5%)
PHF6	4 (9.5%)
WT1	4 (9.5%)
ASXL1	3 (7.1%)
IDH1	3 (7.1%)
PTPN11	3 (7.1%)
SRSF2	2 (4.8%)
JAK2	2 (4.8%)
EZH2	1 (2.4%)
STAG2	1 (2.4%)
CSF3R	1 (2.4%)

Data presented as No. (%) unless otherwise stated

The ORR (CR + CRi + PR) was 92.9% (39/42 patients; 95% CI, 91.6–94.1) after one cycle of the induction regimen, and the composite complete remission rate (CR + CRi) was 90.5% (37 CR, 1 CRi; 95% CI, 89.3–91.6). According to the ELN prognostic criteria (2022), the CR + CRi was 94.1% in favorable-risk group (16/17; 95% CI, 91.5–96.7), 83.3% in intermediate-risk group (5/6; 95% CI, 64.2–100), and 89.5% in adverse-risk group (17/19; 95% CI, 85.5–93.5) (Table 2). Application of Voriconazole or posaconazole and oral Ven (200 mg) on Day 1 did not influence the CR (all patients achieved CR). One patient died before response evaluation. Two NR patients after the induction treatment, one with NRAS, WT1, and TET2 mutations was withdrawn from the clinical trial and achieved CR after salvage therapy; the other patient presented with RUNX1-RUNXT1/t (8;21) received salvage treatment with Ven plus AZA + HA and achieved CR, then he continued the consolidation therapy. The PR patient achieved CR with reinduction with Ven + DA. For patients who achieved CR or CRi after one cycle of induction treatment, MRD was evaluated in 33 patients by flow cytometry; 29 patients attained MRD negativity (87.9%; 95% CI, 84.9–90.8). Of the 11 core-binding factor AML patients, the quantities of fusion genes reduced 100-fold (< 0.1%) in two patients after one cycle of the induction regimen.

**Table 2:** Response Assessment

	Overall (n = 42)	Favorable risk (n = 17) *	Intermediate risk (n = 6) *	Adverse risk (n = 19) *
Overall response rate (CR + CRi + PR)	92.9% (91.6–94.1) [39]	94.1% (91.5–96.7) [16]	83.3% (64.2–100) [5]	94.7% (92.7–96.7) [18]
Composite complete remission rate	90.5% (89.3–91.6) [38]	94.1% (91.5–96.7) [16]	83.3% (64.2–100) [5]	89.5% (85.5–93.5) [17]
CR	88.1% (37/42)	94.1% (16/17)	83.3% (5/6)	84.2% (16/19)
CRi	2.4% (1/42)	0	0	5.3% (1/19)
PR	2.4% (1/42)	0	0	5.3% (1/19)
Died	2.4% (1/42)	0	0	5.3% (1/19)
MRD (-) after induction by flow cytometry	87.9% (95% CI 84.9–90.8; 29/33)			
Responders that received allo-HSCT	2/41(4.9%)			
Time to blood cell count recovery after induction, days				
Time to absolute neutrophil count ≥ 0.5 × 10^9^/L, days	13 (5–26)			
Time to absolute platelet count ≥ 30 × 10^9^/L, days	12 (8–26)			
Even free survival,				
Median, months	NR	NR	NR	NR
12-month, % (95% CI)	82.7% (95% CI: 79.4–86.1%)	88.9% (95%CI: 83.7–94.2)	100%	70.3% (95%CI: 64.7–76.0)
Overall survival				
Median, months	NR	NR	NR	NR
12-month, % (95% CI)	83.1% (95% CI: 78.8–87.4%)	87.5% (95%CI: 81.7–93.4)	100%	70.7% (95%CI: 62.2–79.2)

* = Prognostic stratification according to European Leukemia Net 2022 risk category

After achieving CR or CRi, eight patients withdrew from the clinical trial (four due to financial reasons, one due to infection, one continued treatment at another hospital, one patient showed NR received salvage therapy in another hospital and one died). Thirty-four patients entered consolidation therapy. Up to January 30, 2023, two (cost) and one (prolonged myelosuppression) patient withdrew from the study after 1 and 2 cycles of consolidation therapy, respectively. Moreover, 1 patient received maintenance therapy after three courses of consolidation treatment due to pneumonia, 2 patients underwent allo-HSCT, 20 patients were still on consolidation therapy, and 9 patients entered maintenance therapy (Fig. 2).

**Fig. 3:**
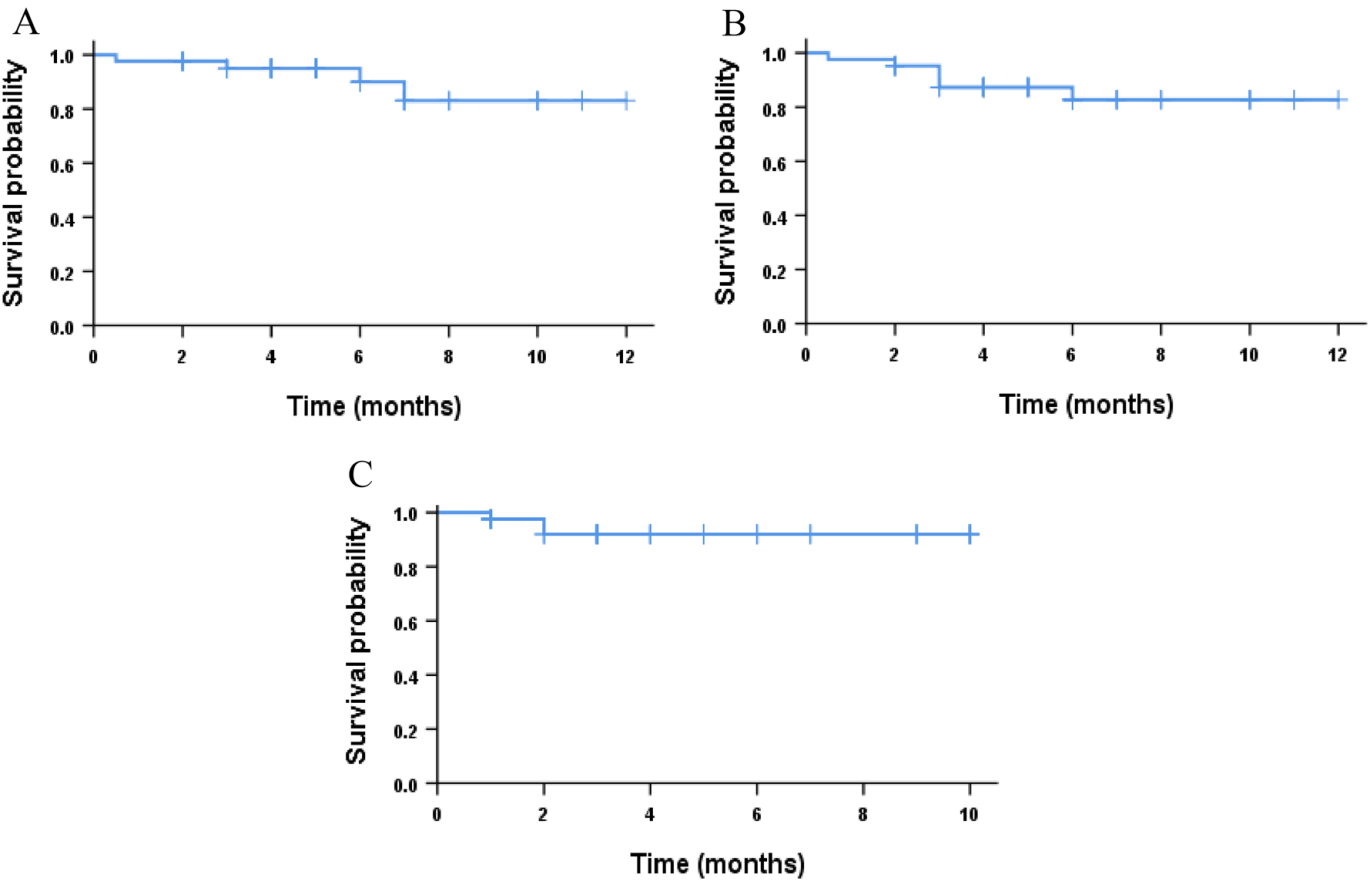
Kaplan–Meier plots of OS, EFS and DFS **A** Overall survival (OS) of all patients. **B** Event free survival (EFS) of all patients. **C** disease-free survival (DFS)

**Fig. 4:**
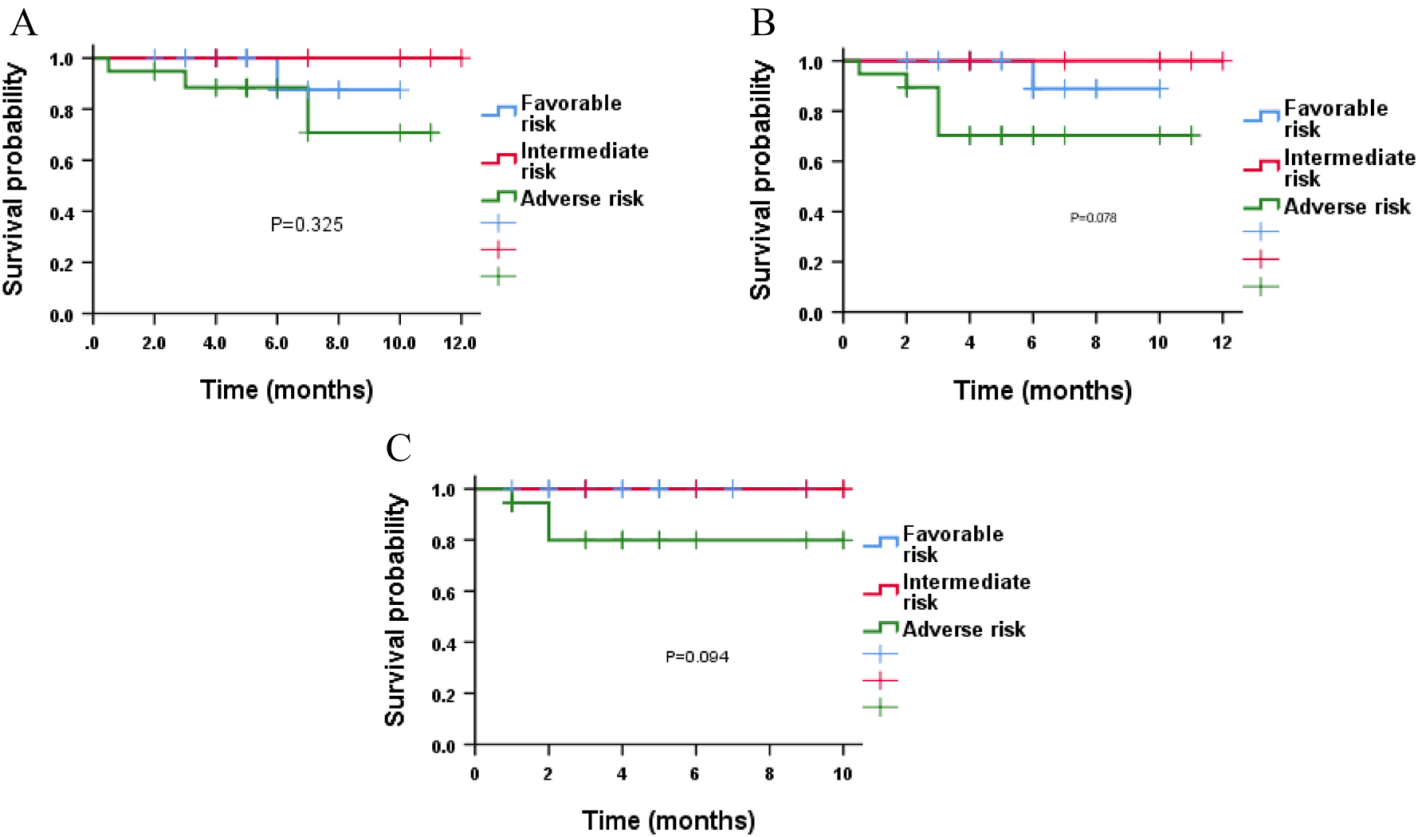
Kaplan–Meier plots of overall survival (OS), event-free survival (EFS) and disease-free survival (DFS) by ELN (2022) prognostic risk **A** OS. **B** EFS. **C** DFS

Until January 30, 2023, with a median follow-up of 5.0 (0.5–12) months, 3 (7.3%) of the 41 patients who had response relapsed, and 4 patients (9.5%) died. Out of the four died patients, one died during induction therapy, one died of relapse after allogeneic transplantation, and two patients withdrawn from the clinical trial and died afterward (one because of infection, the other due to complications during treatment at another hospital). The median OS, EFS, and DFS were not reached, and the estimated 12-month OS, EFS, and DFS rates were 83.1% (95% CI, 78.8–87.4%), 82.7% (95% CI, 79.4–86.1%), and 92.0% (95% CI, 89.8–94.3%), respectively (Fig. 3). After stratifying the patients according to the ELN prognostic risk classification (2022), the estimated 12-month OS, EFS, and DFS rates were 87.5% (95% CI, 81.7–93.4), 88.9% (95% CI, 83.7–94.2), and 100%, respectively, in favorable-risk group; 100% for all three endpoints for moderate-risk patients; and 70.7% (95% CI, 62.2–79.2), 70.3% (95% CI, 64.7–76.0), and 79.9% (95% CI, 74.7–85.2%), respectively for adverse-risk patients (Fig. 4).

Grade 3 to 4 anemia, thrombocytopenia, and neutropenia were the most frequent hematological adverse events during the induction treatment and occurred in all patients (Table 3). In patients who achieved response (CR + CRi + PR), the median time of the absolute neutrophil count recovered to ≥ 0.5 × 10^9^/L and the platelet count to ≥ 30 × 10^9^/L after induction therapy was 13 (range: 5–26) days and 12 (range: 8–26) days, respectively (Table 2).

**Table 3:** Most common adverse events (Grade ≥ 3) during induction

AE	Grade ≥ 3
Haematological adverse events	
Neutropenia	100% (41/41)
Anaemia	100% (41/41)
Thrombocytopenia	100% (41/41)
Non-haematological adverse events	
Febrile neutropenia	92.7% (38/41)
Pneumonia	46.3% (19/41)
Upper respiratory infection	31.7% (13/41)
Skin and soft tissue infection	14.6% (13/41)
Bacteremia	7.3% (3/41)
Intestinal infection	4.9% (2/41)

The most frequent grades 3–4 nonhematological adverse events were febrile neutropenia (38/41 patients, 92.7%), pneumonia (19/41, 46.3%), upper respiratory infections (13/41, 31.7%), bacteremia (3/41, 7.3%), intestinal infections (2/41, 4.9%), and skin and soft tissue infections (6/41, 14.6%) (Table 3). Tumor lysis syndrome was not observed. One patient died because of hemoptysis without pneumonia. The 30-day mortality rate among all patients was 2.4%.

## Discussion

Recently, combination of small-molecule targeted drugs (sorafenib, midostaurin, gilteritinib, enasidenib, ivosidenib, and Ven) with Ven has improved the therapeutic effect of AML especially in patients who were ineligible for standard induction therapy [21–25]. Ven combined with azacytidine or decitabine has been approved as the new standard treatment for older or ineligible patients with newly diagnosed AML [9]. In patients who can tolerate chemotherapy, Ven combined with intensive chemotherapy achieves high CR + CRi and MRD-negative rates [12–15]. However, severe and prolonged myelosuppression remains a major concern, and most patients still need allo-HSCT. Hence, shortening the myelosuppressive period and enabling patients to complete their treatment regimen are essential for those who cannot undergo allo-HSCT.

DAV regimen was reported by Wang HF in 2022. The composite CR rate after one cycle of the DAV regimen was 91%, and 29 (97%) of 30 patients who reached complete remission had undetectable MRD [12]. CR + CRi rate was 94% in patients who were treated with Ven + CLIA, and 82% of patients had undetectable MRD [14]. In our trial, the induction regimen of Ven combined with dose-reduced DA (2 + 6) achieved a high composite CR rate of 90.4% (CR 37/42, CRi 1/42) and MRD-negative rate of 87.9% (29/33) in patients who reached complete remission after one cycle of induction therapy. The ORR, composite CR, and MRD-negative rates in our trial were similar to those in previous reports. Notably, the myelosuppressive period of our induction regimen appears to be the shortest than Ven combinations with intensive chemotherapy (DA, IA, CLIA, FLAG-IDA) reported before, with a median of 13 and 12 days for neutrophil and platelet recovery, respectively, after induction therapy [12–15]. The duration of the myelosuppressive is similar to that of conventional chemotherapy (DA or IA).

To the best of our knowledge, our therapeutic regimen is the first to combine Ven with ID-Ara-c as consolidation therapy. Based on our previous studies, two combinations of Ven with ID-Ara-c for intensive therapy and three reduced-dose consolidation therapies were designed in the regimen, and clinical data showed that the consolidation therapy was well tolerated. As of January 30, 2023, only one patient had withdrawn from the clinical trial because of the prolonged myelosuppressive period after the first consolidation therapy. Compared with previously reported data [13–15], this consolidation treatment regimen showed good tolerability, as there was no significant prolongation of the myelosuppressive period, allowing most of the patients to complete the regimen. Until the follow-up date, nine patients had initiated maintenance therapy.

Most of our patients were unable to undergo allo-HSCT, we added danazol and thalidomide into the maintenance therapy to improve both RFS and OS. The possible mechanisms are: (1) androgens can inhibit the proliferation of leukemic cell lines and induce differentiation [26]; maintenance therapy with norethandrolone can improve survival in elderly patients with AML [27]; (2) as an androgen, danazol can stimulate hematopoiesis, which may reduce the incidence of hemocytopenia, and it can also improve symptoms (such as fatigue); and (3) previous studies have shown that thalidomide plays a role of anti-cell proliferation, induction of apoptosis, blockage of tumor–stroma interactions, and induction of changes in immune responses. Until the date of follow-up, 9 patients entered maintenance therapy, none discontinued maintenance therapy due to myelosuppression or intolerance, and none relapsed. Because the number of patients who entered maintenance therapy is small, and the long-term outcome of maintenance therapy cannot be observed in short period of this study. Next, we will conduct a clinical randomized controlled trial to investigate whether the maintenance therapy could improve the outcome in the future according to the conclusion after a longer follow-up times.

Of the 42 enrolled patients, 11 patients are core-binding factor AML [10 patients with RUNX1-RUNXT1/t (8;21) and 1 with CBFB/MYH11/inv (16)]. Out of them, six patients had kit mutations, and three had FLT3/ITD. The CR rate after one cycle induction therapy was 90.9% (10/11). The NR patient achieved CR after salvage treatment with Ven plus AZA and HA. Nevertheless, qPCR analyses revealed 3-log reduction (≤ 0.1) in RUNX1-RUNX1T1 or CBFB/MYH11 fusion transcript level after one cycle of induction therapy in only two patients. That means although the CR rate was high, the remission depth was not ideal.

Until January 30, 2023, with a median follow-up of 5 months, the median OS, EFS, and DFS had not reached, and the estimated 12-month OS, EFS, and DFS rates were 83.1% (95% CI, 78.8–87.4), 82.7% (95% CI, 79.4–86.1), and 92.0% (95% CI, 89.8–94.3), respectively. The primary cause of relapse or death was patient withdrawal from clinical trials, as previously described. Considering that the proportion of patients who received allo-HSCT (4.8%) in this clinical trial was lower than that in previous reports, this result is encouraging. The results indicate that Ven combined with intense chemotherapy may improve the long-term efficacy of AML patients. With more patients enrolled and the follow-up period extended, we will further explore the types of AML may benefit from this regimen.

Of the 42 patients, only 1 died during induction because of hemoptysis without pneumonia. The 30-day mortality rate for all patients was 2.4%. The most frequent grades 3–4 non-hematological adverse events were infections, including febrile neutropenia, pneumonia, upper respiratory infections, bacteremia, intestinal infections, and skin and soft tissue infections. None tumor lysis syndrome was observed. Therefore, this regimen is safe and well tolerated.

This study still has some limitations. The number of patients enrolled was small and the duration of the follow-up period was short. Consequently, it is necessary to continuously enroll more patients and extend the follow-up period to further verify the current conclusions about this regimen.

In summary, the combination of Ven with DA (2 + 6) is an well tolerated and highly effective regimen for patients with de novo AML. A larger number of patients and an extended follow-up period are warranted to confirm these findings.

## Supplementary Information

**Additional file 1: **Compared high-dose cytarabine with Ven combined with intermediate-dose cytarabine shown that the duration of myelosuppression, safety and efficacy were similar.

**Additional file 2.** Chemotherapy regimen;

**Additional file 3.** Inclusion and Exclusion criteria;

**Additional file 4.** The calculation of the sample size.

## Data Availability

The datasets used during the current study are available from the corresponding author on reasonable request. We encourage investigators interested in data sharing and collaboration to contact the corresponding author.
