# Supplementary material for: Venetoclax combined with daunorubicin and cytarabine (2 + 6) as induction treatment in adults with newly diagnosed acute myeloid leukemia: a phase 2, multicenter, single-arm trial
Source: Exp Hematol Oncol. 2023 May 12;12:45. doi: 10.1186/s40164-023-00409-y (PMC10176670; doi:10.1186/s40164-023-00409-y)
Supplement: Supplementary file 1 — Additional file 1: Compared high-dose cytarabine with Ven combined with intermediate-dose cytarabine shown that the duration of myelosuppression, safety and efficacy were similar. [file 40164_2023_409_MOESM1_ESM.docx]

**Appendix：**

The myelosuppression, safety and efficacy between Ven+ID-Ara-c and HD-Ara-c

|  | **Ven+ID-Ara-c**  **(n=23)** | **HD-Ara-c**  **(n=23)** | **Pvalue** |
| --- | --- | --- | --- |
| **Sex**（male/female） | 12/11 | 11/12 | 1.0 |
| **Age, years** Median (IQR) | 54（27-63） | 44（18-59） | 0.09 |
| **MRD by flow cytometry**  Before treatment  After treatment | 3/20 | 1/22 | 0.608  1.0 |
|  | 2/20 | 1/22 |  |
| **Before treatment**： |  | | |
| WBC (× 10^9^/L) | 4.98(1.78-9.27) | 4.49(2.62-9.83) | 0.81 |
| Plt (× 10^9^/L)  **During treatment** | 216(84-804) | 341(98-1246) | 0.042 |
| Nadir of WBC (× 10^9^/L) | 0.19(0.06-0.61) | 0.27(0.01-1.11) | 0.116 |
| Nadir of neutrophil count (× 10^9^/L) | 0.01（0-0.05） | 0.01（0-0.52） | 0.019 |
| Nadir of platelet count | 5（1-38） | 3（1-18） | 0.062 |
| Mean duration of leukopenia, day (range)  (<1× 10^9^/L) | 8（6-14） | 7（0-18） | 0.542 |
| Mean duration of neutropenia, day (range)  (<0.5× 10^9^/L) | 7（6-12） | 7（0-16） | 0.684 |
| Mean duration of thrombocytopenia, day  (<30× 10^9^/L) | 7(1-13) | 8(4-15) | 0.226 |
| Number of platelet transfusions | 1（0-2） | 2（1-6） | <0.001 |
| Number of RBC transfusions（u） | 0（0-4） | 2（0-8） | 0.029 |
| Mean duration of hospital, day (range) | 20（17-23） | 20（17-32） | 0.189 |
