# Supplementary material for: Venetoclax combined with daunorubicin and cytarabine (2 + 6) as induction treatment in adults with newly diagnosed acute myeloid leukemia: a phase 2, multicenter, single-arm trial
Source: Exp Hematol Oncol. 2023 May 12;12:45. doi: 10.1186/s40164-023-00409-y (PMC10176670; doi:10.1186/s40164-023-00409-y)
Supplement: Supplementary file 2 — Additional file 2. Chemotherapy regimen; [file 40164_2023_409_MOESM2_ESM.docx]

**Chemotherapy regimen**

**Induction regimen** (Venetoclax(ven) combined with daunorubicin and cytarabine)

Ven 400 mg, days 1–7

Daunorubicin 60 mg/m², days 2–3

Cytarabine 100 mg/m^2^/q12 h, days 2–7

**Consolidation therapy**

I Ven 400 mg, days 1-7,

Cytarabine 1g/m^2^/q12h, days 2-4

II Ven 400 mg, days 1-7,

Cytarabine 1g/m^2^/q12h, days 2-4

III Daunorubicin 60 mg/m², days 1–2

Cytarabine 100 mg/m²/q12h, days 1-5

IV Homoharringtonine 2 mg/m², days 1–5

Cytarabine 100 mg/m²/q12h, days 1-5

V Homoharringtonine 2 mg/m², days 1–5

Cytarabine 100 mg/m²/q12h, days 1-5

**maintenance theray**

{VEN combined with azacitidine (AZA), danazol (DNZ) and thalidomide (THD)}

VEN 200 mg, days 1-7,

AZA 100 mg, days 1-5,

DNZ 200 mg po bid, days 8-28

THD 100 mg po qd, days 8-28

Maintenance was permitted on D1-28 every month for at least six cycles in patients not proceeding to stem-cell transplantation. After stoping venetoclax and azacytidine, patients continued the DNZ and THD maintenance therapy for up to 3 years unless relapse or intolerance occurred.
