# Supplementary material for: Venetoclax combined with daunorubicin and cytarabine (2 + 6) as induction treatment in adults with newly diagnosed acute myeloid leukemia: a phase 2, multicenter, single-arm trial
Source: Exp Hematol Oncol. 2023 May 12;12:45. doi: 10.1186/s40164-023-00409-y (PMC10176670; doi:10.1186/s40164-023-00409-y)
Supplement: Supplementary file 3 — Additional file 3. Inclusion and Exclusion criteria; [file 40164_2023_409_MOESM3_ESM.doc]

**Inclusion criteria：**

Subjects suitable for this study must meet all the following criteria.

1) Patients with acute myeloid leukemia other than APL that met the FAB criteria;

2) Patients with acute myeloid leukemia other than APL that met World Health Organization diagnostic criteria (WHO 2016);

3) Patients with acute myeloid leukemia met the WHO (2016) criteria “AML with myelodysplasia-related changes” without the history of MDS and/or MPN;

4) Except Acute panmyelosis with myelofibrosis, Myeloid sarcomas and AML not classified separately by WHO criteria;

5) Aged 16 to 60 years old;

6) Eastern Cooperative Oncology Group (ECOG) score 0 to 2;

7) Requirements for passing the following laboratory indicators (performed within 7 days prior to treatment):

i) Total bilirubin ≤ 1.5 times the upper limit of normal value;

ii) Aspartate aminotransferase (AST) and Alanine aminotransferase (ALT)≤ 2.5 times the upper limit of normal value;

iii) Serum creatinine < 2 times the upper limit of normal;

iv) Cardiac enzymes < 2 times the upper limit of normal value;

v) Cardiac ejection fraction was within the normal range tested by Echocardiography (ECHO).

8) Informed consent must be signed by the patients themselves or their immediate family members before the study procedures; if patients under the age of 18, the informed consent signed by legal guardian; if informed consent signed by patient did not conducive to treatment, could be signed by the legal guardian or the patient's immediate family member.

**Exclusion criteria:**

1) Patients who have received induction treatment before, regardless of efficacy.

2) Secondary leukemia (mainly patients belong to the treatment-related AML according to WHO 2016 classification or have a history of MDS / MPN).

3) Patients with other hematological disease (researchers considered ineligible enrolled, such as hemophilia and myelofibrosis, etc. Patients with previous blood abnormalities, but with MDS and MPD excluded from bone marrow examination, were admitted).

4) Accompany with other systemic malignancies (requiring treatment).

5) Pregnant and lactating women. Female subjects of reproductive age must have a negative pregnancy test within 7 days of starting the trial.

6) Abnormal liver and kidney functions exceeding the inclusion criteria.

7) Cardiovascular disease, one or more of the following:

i) uncontrolled or symptomatic angina;

ii) history of myocardial infarction within 6 months;

iii) Arrhythmia need medical treatment or with severe symptoms;

iv) Uncontrolled or symptomatic congestive heart failure (NYHA grade > 2);

v) Cardiac ejection fraction lower than normal range.

8) Subjects with uncontrollable systemic infection (viral, bacterial or fungal)..

9) Those considered unfit for enrolled the trail by the researcher.
