# Supplementary material for: Venetoclax combined with daunorubicin and cytarabine (2 + 6) as induction treatment in adults with newly diagnosed acute myeloid leukemia: a phase 2, multicenter, single-arm trial
Source: Exp Hematol Oncol. 2023 May 12;12:45. doi: 10.1186/s40164-023-00409-y (PMC10176670; doi:10.1186/s40164-023-00409-y)
Supplement: Supplementary file 4 — Additional file 4. The calculation of the sample size. [file 40164_2023_409_MOESM4_ESM.doc]

**Statistical analysis:**

The calculation of the sample size：

Sample size was determined according to minimax Simon's two-stage design, with a composite complete remission rate of lower than 60% for the null hypothesis, which would indicate that the activity of the regimen was unacceptable, and a response rate of higher than 80% for the alternative hypothesis, which would indicate that the regimen deserved further exploration. We calculated a sample size that would give power of 80%, at a significance level of 5%, to test the null and alternative hypotheses regarding the primary endpoint. In the first stage, 13 participants would be needed, with an additional 22 participants in the second stage after the interim analysis. With 13 participants in the first stage, the trial would be discontinued if 8 or fewer responded to the treatment. If the trial continued to the second stage, the treatment activity would be rejected if 25 or fewer of the 35 participants responded to the treatment. If the number who responded was greater than 25 participants, the null hypothesis would be rejected. Accounting for a 10% dropout rate, the estimated final sample size was 39 patients (35 patients who could be evaluated).
